# Surgical Repair of Late Complications in Patients Having Undergone Primary Hypospadias Repair during Childhood: A New Perspective

**DOI:** 10.1155/2012/705212

**Published:** 2012-04-10

**Authors:** Guido Barbagli, Salvatore Sansalone, Rados Djinovic, Massimo Lazzeri

**Affiliations:** ^1^Center for Reconstructive Urethral Surgery, 52100 Arezzo, Italy; ^2^Sava Perovic Foundation, 11000 Belgrade, Serbia

## Abstract

*Background*. The repair of complications in patients who had undergone hypospadias repair is still an open problem. *Patients and Methods*. We conducted a retrospective study of patients treated for late complications after hypospadias repair. Study inclusion criteria were patients presenting urethral, corpora cavernosa deformity, and/or penile defects due to previous hypospadias repair. Exclusion criteria were precancerous or malignant lesions and incomplete data on personal medical charts. Preoperative evaluation included clinical history, physical examination, urine culture, residual urine measurement, uroflowmetry, urethrography, urethral sonography, and urethroscopy. The patients were classified into four different groups. Success was defined as a normal functional urethra, with apical meatus, no residual penile curvature or esthetic deformity of the genitalia. *Results*. A total of 1,176 patients were entered in our survey. Out of the 1,176 patients, 301 patients (25.5%) underwent urethroplasty (group 1), 60 (5.2%) corporoplasty (group 2), 166 (14.1%) urethroplasty and corporoplasty (group 3), and 649 (55.2%) complex genitalia resurfacing (group 4). Mean followup was 60.4 months. Out of the 1,176 cases, 1,036 (88.1%) were considered successful and 140 (11.9%) failures. *Conclusion*. The majority of patients (55.2%) with failed hypospadias repair require surgical reconstruction to fully resurfacing the glans and penile shaft.

## 1. Introduction

The surgical repair of primary hypospadias in childhood may result in late postoperative complications involving the external urinary meatus (stenosis and retrusive meatus), the urethra (stricture, fistula, and diverticulum), the corpora cavernosa (penile curvature, torsion, or deformity), the preputial skin, or the genitalia [1–5]. These complications may involve a single compartment of the male genitalia (urethra, corpora cavernosa, glans, or penile or scrotal skin), or a combination of them. The main causes of these late surgical complications are poorly executed procedures, postoperative infection, wound dehiscence, urine extravasation, hematoma, or ischemia or necrosis of transplanted tissues [1–3]. However, hypospadias repair may also fail many years after achieving successful functional and cosmetic results by primary repair, and a urethral stricture may develop decades after the initial hypospadias surgery [3].

The surgical approach to failed hypospadias repair is mainly focused on urethral reconstruction and rarely on problems involving the corpora cavernosa or the complete resurfacing of the genitalia [4, 5]. Our experience with the largest series of patients with failed hypospadias repair published in the literature to date provided us with a new perspective on this difficult problem [6, 7]. Patients with complications after primary hypospadias repair represent a nonhomogeneous population, presenting a wide range of surgical problems as well as numerous surgical challenges. For this reason, we classified patients presenting with failed hypospadias repair into different groups according to the involvement of a single versus multiple anatomical compartment(s) of the genitalia and according to the complexity of surgical reconstruction.

## 2. Methods

This is an observational, descriptive, and retrospective study on patients treated for late complications after primary hypospadias repair. Study inclusion criteria were patients presenting urethral pathological conditions, corpora cavernosa deformity, and/or penile and genitalia defects due to previous hypospadias surgery. Exclusion criteria were precancerous or malignant penile lesions, incomplete data on personal medical charts, or any condition that would interfere with the patient's ability to provide an informed consent.

Patients were classified into four different groups according to the involvement of a single or multiple anatomical compartment(s) of the genitalia at the time of surgery. Group 1 included patients requiring urethroplasty for urethral pathological conditions; group 2 included patients requiring corporoplasty for deformity of the corpora cavernosa; group 3 included patients requiring urethroplasty for urethral pathological conditions associated with corporoplasty for deformity of the corpora cavernosa; group 4 included patients requiring complex reconstructive procedures including urethra, corpora cavernosa, and glans and penile skin resurfacing.

Preoperatively, all patients underwent urine culture, abdominal ultrasonography, retrograde and voiding urethrography, and urethroscopy using a 7 Fr. rigid urethroscope. Photographs of the penis in full erection were used to investigate patients with glans or penile curvature or torsion. Evaluations were scheduled at 3, 6, and 9 months postoperatively, and then annually thereafter. At followup, patients underwent a physical examination and uroflowmetry. When symptoms of decreased force of stream were present, and uroflowmetry was less than 12 mL per second, meatal calibration, urethrography, ultrasonography, and urethroscopy were repeated. Patients presenting with residual curvature were again asked to supply photographs of the penis in full erection. Success was defined as a functional urethra without fistula, stricture, or residual chordee and a glandular meatus with cosmetically acceptable genitalia. The need for meatal or urethral dilation and complications or a poor cosmetic appearance of the genitalia requiring revision was considered a failure. The primary outcome measure was the evaluation of the success rate of surgical procedures, and the secondary end point investigated the difference in success rate between the different surgical procedures.

## 3. Results

From 1988 to 2007, a total of 1,176 patients from our two centers were entered in our survey according to the inclusion/exclusion criteria. Nine hundred fifty-three (81%) of the patients were evaluated in Serbia and 223 (19%) in Italy. Mean patient age was 31 years (range from 1 to 76).

The age of patients at the time of the primary surgery was from 1 to 12 years, but patient stratification by age was not possible. The average time from primary surgery to the time of complication repair was not possible to certainly summarize. Although some patients could not indicate the exact site of the primary original meatus, including those who underwent several operations many years before our observation, intraoperative findings allowed us to establish exactly where the meatus had originated. The site of original hypospadias was glanular in 193 cases (16.4%), penile in 702 (59.7%), and penoscrotal in 281 (23.9). The type of late complications after primary repair of hypospadias was not related to the site of the original meatus. Mean followup was 65 months.

Group 1 included 301 patients (25.5%) requiring urethroplasty for urethral pathological conditions (meatal, penile or bulbar stricture, retrusive meatus, fistula, and diverticulum), of which 162 (53.8%) underwent one-stage repair and 139 (46.2%) multistage repair. Group 2 included 60 patients (5.2%) requiring corporoplasty for deformity of the corpora cavernosa (residual penile curvature, corpora cavernosa deformity, and/or penile shortening or torsion), of which 38 (63.2%) underwent Nesbit corporoplasty, 14 (23.4%) plication of the corpora, and 8 (13.4%) corporoplasty using graft material. Group 3 included 166 patients (14.1%) requiring urethroplasty for urethral pathological conditions associated with corporoplasty for deformity of the corpora cavernosa (urethral stricture, fistula, diverticulum, and residual glans/penile curvature). These patients underwent urethroplasty and corporoplasty using a combination of the techniques used in groups 1 and 2.

Group 4 included 649 patients (55.2%) requiring complex reconstructive procedures, including urethra, corpora cavernosa, glans, and penile skin resurfacing (glans dehiscence, partial glans necrosis, glans torsion or curvature, loss of penile or scrotal skin, midline septum, penile skin torsion, abnormal penoscrotal or penopubic junction, buried penis, trapped penis, and other). These patients underwent nonstandard combined procedures to repair urethral, corpora cavernosa, and complex genitalia deformity.

Out of the 1,176 cases, 1,036 (88.1%) were classified as successful, and 140 (11.9%) were considered failures. The success rate was 89.7% (group 1), 96.7% (group 2), 88.5% (group 3), and 86.4% (group 4), respectively (Table 1).

## 4. Discussion

Surgical treatment of patients with late complications after hypospadias surgery represents a complex problem, as this difficult population of patients has been left with deformities that fully involve the genitalia and are significantly worse than the simple primary congenital hypospadias [8–10]. Our present survey shows that reoperative surgery in 55% of patients involves not only the urethra, but requires complete resurfacing of the genitalia. Patients with complications after primary hypospadias repair represent a nonhomogeneous population presenting a wide range of surgical problems as well as numerous surgical challenges according to the complexity of reconstruction each providing a different outcome.

In our survey, patients requiring corporoplasty for residual deformity of the corpora cavernosa (group 2) show the highest success rate (96.7%), and the majority of these patients were treated using standard techniques (63.2% Nesbit and 23.4% corpora plication). In this group, only 8 cases (13.4%) required non-conventional corporoplasty using graft material as in surgery for Peyronie's disease. Patients requiring urethroplasty for meatal, penile or bulbar strictures, retrusive meatus, fistula, and diverticulum (group 1) show an 89.7% success rate, and all of these patients were treated using standard techniques currently suggested in the literature, mainly using oral mucosa as substitute material in one- or multi-stage procedures, without significant changes in the surgical steps [3, 7, 9, 10]. The success rate decreased (88.5%) in patients requiring urethroplasty in association with corporoplasty (group 3) because the surgical reconstruction completely involved the penile shaft (urethra and corpora cavernosa). These patients required different surgical techniques or a combination of them with a more aggressive approach to fully expose the urethra and corpora cavernosa. Finally, in patients presenting complex genitalia deformity (group 4), the success rate was lower (86.4%), and these patients underwent non-conventional combined surgical procedures.

Our present survey on a large series of patients allowed us to gain a new perspective on surgical repair of late complications in patients who had undergone primary hypospadias repair during childhood. We realize that failed hypospadias repair may consist of defects of single compartments (urethra, corpora cavernosa, glans, or penile and scrotal skin) of the genitalia or a combination of them, and the surgical complexity depends on the number of compartments involved. In our series, reoperative surgery was restricted to the urethra in only 25.5% of the cases and to the corpora cavernosa in only 5.2% of the cases. These patients (groups 1 and 2) did not require any special expertise because the majority of them were treated using oral mucosa graft or corporoplasty according to well-known standard techniques reported in the literature and which are reliable in the hands of any surgeon from any urology department. The success rate in these patients is high. Whereas, patients presenting urethral complications associated with corpora cavernosa deformity (group 3) represent a more difficult population to treat and require greater experience in reconstructive urological procedures of the genitalia, which should not be attempted by the inexperienced surgeon but instead referred to specialized centers. Patients requiring complete and complex resurfacing of the genitalia (group 4) should also be referred to specialized centers.

In Table 2 we summarized a new algorithm for surgical repair of late complications in patients having undergone primary hypospadias repair during childhood (Table 2). In recent years, the sexual, psychological, and emotional value of the sexual organ has changed. It is common knowledge that a normal esthetic appearance of the penis contributes positively to the patient's self-esteem, body image, and sexuality. Thus, it is imperative that the wishes of patients wanting to achieve normal function and an appearance of the genitalia after reoperative surgery for failed hypospadias repair should be considered a priority. Reconstructive surgery of the male genitalia should allow penis anatomy and function to remain as close as possible to the physiological situation, with normal cosmetic appearance, penile length, and straight shaft, allowing the patient to feel fully satisfied with his self-image and genitalia integrity.

Now is the time to develop a perspective on problems involving patients having undergone primary hypospadias repair during childhood. These patients had undergone numerous operations to repair urethral and penile defects without a satisfactory outcome and sometimes prefer waiting for improvements in surgery which might offer better results than in previous years. The question is “are urologists now more able to offer better results than many years ago with regards to the esthetic reconstruction of the corpora cavernosa and genitalia?” It will be possible in the near future, but now it is essential to establish centers specialized in treatment of these patients, where collaboration between the urologist and the surgeon who is widely skilled in reconstructive surgery of the corpora cavernosa (penile prosthesis implantation, surgery for Peyronie's disease, and surgery for male to female transition) can ensure the best functional and esthetic outcome. For example, in our present experience, some patients under repair corpora cavernosa deformity required complex techniques using substitute graft materials such as in patients with severe Peyronie's disease.

Should not patients with complex failed hypospadias repair be referred to a specialized center of expertise [11]? Medically and ethically speaking, it is the right thing to do. Our study does present some important weaknesses as it is only an observational, descriptive, and retrospective survey of patients treated for complications after hypospadias repair. We were unable to investigate and compare postoperative urinary and sexual complications or sequelae using specific patient-reported outcome measures (PROMs) questionnaire in the four groups. We also did not investigate health-related quality of life (HRQoL) in our groups.

Recently, Jackson et al. [12] defined a succinct, practical, and psychometrically robust validated PROM designed specifically to quantify changes in voiding symptoms and following urethral stricture surgery. This questionnaire was also validated in Italian language by Barbagli et al. [13]. We sincerely hope that our preliminary study will open the way to further studies that include more detailed data. With international validation of new questionnaires specifically dedicated to patients who have undergone urethroplasty [12, 13], new larger studies are more than welcome.

Our study has several weaknesses, including the difficulty to collecting data on all patients covering all aspects of hypospadias history and subsequent repairs and the fact that only two centers were involved. Also, our sample may not reflect the general American or European situation. Furthermore, we failed to obtain information on patient's quality of life and satisfaction after surgery.

In some patients, we were unable to investigate and extrapolate some important data from the medical charts of patients as we report in Section 3. Mean patient age was 31 years, and the majority of patients had undergone a variety of hypospadias repair in the past, ranging from one to more than eight procedures. Some patients often lost medical charts related to these operations and were not able to indicate whether the surgical technique used for the repair was a one-stage or multistage procedure. Moreover, at the beginning of our work, end of 80s, databases were not available in most of Italian hospitals as well as in Serbia, and only recently databases are fully available.

In our populations, was not possible it/to certainly quantify and summarize the incidence of early postoperative complications after primary hypospadias repair. Only in few patients it was possible to establish that primary hypospadias repair was surely complicated by early complications. The majority of these patients had undergone numerous operations to repair these postoperative complications without a satisfactory outcome and sometimes prefer waiting many years before accepting a new surgical repair for a serious psychological involvement or hoping for improvements in surgery which might offer better results than in previous years.

## 5. Conclusions

Surgical repair of late complications in patients having undergone primary hypospadias repair during childhood still represents a challenging problem. In the majority of patients, repair of these deformities requires full collaboration between the urologist and the surgeon who has developed vast experience in plastic and reconstructive surgery of the genitalia.

## Figures and Tables

**Table 1:** Success rate according to the complication and repair.

Group no. of patients	Type of complications	Type of repair	Success rate %
Group 1 301 (25.5%)	Urethral stricture, fistula, diverticulum, retrusive meatus, and/or other	Urethroplasty	270 (89.7%)
Group 2 60 (5.2%)	Residual penile curvature corpora cavernosa deformity, penile shortening or torsion, and/or other	Corporoplasty	58 (96.7%)
Group 3 166 (14.1%)	Stricture, fistula, diverticulum associated with residual glans or penile curvature or deformity, and/or other	Urethroplasty corporoplasty	147 (88.5%)
Group 4 649 (55.2%)	Glans dehiscence, glans necrosis, glans torsion or curvature, loss of penile/scrotal skin, midline septum, abnormal penoscrotal or penopubic junction, buried penis, trapped penis, other	Genitalia resurfacing	561 (86.4%)

**Total**			**1036 (88.1%)**
**1,176**			

**Table 2:** Algorithm for surgical repair.

Group	Type of repair	Difficulty in surgery	Place of repair
Group 1	Urethroplasty	Standard	Urological department
Group 2	Corporoplasty	Standard	Urological department
Group 3	Urethroplasty Corporoplasty	High	Specialized referral center
Group 4	Genitalia resurfacing	Superior	Specialized referral center
